# Gene expression profiles and neural activities of Kenyon cell subtypes in the honeybee brain: identification of novel ‘middle-type’ Kenyon cells

**DOI:** 10.1186/s40851-016-0051-6

**Published:** 2016-07-29

**Authors:** Kumi Kaneko, Shota Suenami, Takeo Kubo

**Affiliations:** Department of Biological Sciences, Graduate School of Science, The University of Tokyo, Bunkyo-ku, Tokyo, 113-0033 Japan

**Keywords:** Honeybee, Social behavior, Foraging, Brain, Mushroom body, Kenyon cell, *mKast*, Arrestin domain-containing protein, Neural activity mapping, Hymenopteran insect

## Abstract

In the honeybee (*Apis mellifera* L.), it has long been thought that the mushroom bodies, a higher-order center in the insect brain, comprise three distinct subtypes of intrinsic neurons called Kenyon cells. In class-I large-type Kenyon cells and class-I small-type Kenyon cells, the somata are localized at the edges and in the inner core of the mushroom body calyces, respectively. In class-II Kenyon cells, the somata are localized at the outer surface of the mushroom body calyces. The gene expression profiles of the large- and small-type Kenyon cells are distinct, suggesting that each exhibits distinct cellular characteristics. We recently identified a novel gene, *mKast* (*middle-type Kenyon cell-preferential arrestin-related gene-1*), which has a distinctive expression pattern in the Kenyon cells. Detailed expression analyses of *mKast* led to the discovery of novel ‘middle-type’ Kenyon cells characterized by their preferential *mKast*-expression in the mushroom bodies. The somata of the middle-type Kenyon cells are localized between the large- and small-type Kenyon cells, and the size of the middle-type Kenyon cell somata is intermediate between that of large- and small-type Kenyon cells. Middle-type Kenyon cells appear to differentiate from the large- and/or small-type Kenyon cell lineage(s). Neural activity mapping using an immediate early gene, *kakusei*, suggests that the small-type and some middle-type Kenyon cells are prominently active in the forager brain, suggesting a potential role in processing information during foraging flight. Our findings indicate that honeybee mushroom bodies in fact comprise four types of Kenyon cells with different molecular and cellular characteristics: the previously known class-I large- and small-type Kenyon cells, class-II Kenyon cells, and the newly identified middle-type Kenyon cells described in this review. As the cellular characteristics of the middle-type Kenyon cells are distinct from those of the large- and small-type Kenyon cells, their careful discrimination will be required in future studies of honeybee Kenyon cell subtypes. In this review, we summarize recent progress in analyzing the gene expression profiles and neural activities of the honeybee Kenyon cell subtypes, and discuss possible roles of each Kenyon cell subtype in the honeybee brain.

## Background

**Fig. 1:**
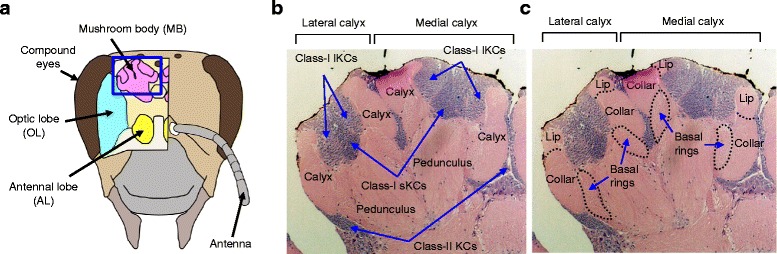
Structure of the honeybee brain and mushroom bodies. **a** Schematic drawing of the honeybee brain. MB, mushroom body; OL, optic lobe; AL, antennal lobe. **b** Hematoxylin-eosin staining of the left MB, which corresponds to the region indicated by a square in panel (**a**). Each MB has two cup-like structures: lateral and medial calyces, each of which comprises calyces and a pedunculus. lKC and sKC, large- and small-type Kenyon cells, respectively. **c** The honeybee MB calyces are subdivided into three layers: lips, collars, and basal rings. Original figure (photo for the hematoxylin-eosin staining of the left MB) from [20] was modified and used for panels (**b**) and (**c**)

In mammals, various advanced brain functions are distributed to distinct areas of the brain [1]. In insects, too, brain areas and functions are closely related. The insect brain contains major structures, such as the mushroom bodies (MBs, a higher-order brain center), antennal lobes (ALs, a primary olfactory and mechanosensory center), optic lobes (OLs, a primary center of visual information), and subesophageal ganglion (SOG, a center for taste and feeding behavior) (Fig. 1a) [2–4]. One of the most intriguing questions in insect neuroscience is how neural circuits regulate their intrinsic behaviors, especially social behaviors. The European honeybee (*Apis mellifera* L.) is a well-known eusocial insect and its behaviors, including dance communication, have been studied extensively [5–7].

Honeybees live in colonies that usually comprise a single queen (the reproductive caste) and several thousands of workers (the labor and non-reproductive caste), and from spring to autumn, several hundreds of drones. Queens and workers are female, and drones are male [5, 6]. Workers exhibit an age-dependent ‘division of labor’; for example, bees 6 to 12d of age take care of the brood inside the hives (nurse bees), whereas older bees (usually >16 d of age) collect nectar and pollen outside their hives (foragers) [5, 6]. After a successful foraging flight, workers (dancers) convey food source location to their nestmates (followers) through dance communication [7]. These social characteristics have long attracted researchers.

Importantly, recent studies suggest some relations between honeybee MB functions and foraging behavior [8–10]. Insect MBs are a paired structure comprising two cup-like calyces, peduncles, and intrinsic neurons called Kenyon cells (KCs), which have distinct subtypes. The MB structures of the honeybee are elaborate compared with those of some other insect species (e.g., fruit fly). Honeybee KCs are categorized into three subtypes based on their morphology; class-I large-type KCs (lKCs or ‘inner non-compact’ KCs), class-I small type KCs (sKCs or ‘inner compact’ KCs), and class-II KCs (or ‘outer compact’ KCs). While the somata of class-I lKCs and sKCs are localized at the edges and inner core of the MB calyces, respectively, the somata of class-II KCs surround the outer surface of the MB calyces (Fig. 1b). KCs extend neurites that branch to form dendrites in the calyces and axons in the pedunculus [2–4]. Axons from the class-I KCs bifurcate to form two distinct lobes, called the medial and vertical lobes, while those from the class-II KCs form single lobe called the gamma lobe. The honeybee MB calyces are subdivided into three layers: the lip, collar, and basal ring (Fig. 1c). The lip receives olfactory information from the ALs, the collar receives visual information from the OLs, and the basal ring receives both olfactory and visual information [2–4, 11–13].

The preferential expression in *Drosophila* MBs of some genes responsible for mutants in learning, such as *dunce*, *rutabaga*, and *DCO*, established the involvements of the MBs in learning and memory in the fruit fly [14–16]. In the honeybee, the MBs are involved in learning and memory, as well as multimodal sensory integration [17–19]. The MB neuropil volume depends on the division of labor of the workers [8], and while the complexity of the MB neuropil increases with age, foraging experience also enhances neuropil outgrowth [9]. A previous study reported that parasitoidism, rather than sociality, is associated with elaboration of the MBs in hymenopteran insect brains, and proposed that the cognitive demands of host-finding behavior in parasitic wasps drove the acquisition of the evolutionarily novel MB architecture before sociality was acquired [10]. How each KC subtype contributes to MB function in honeybee social behaviors, including foraging behavior, however, remains largely unknown.

Various genes are expressed in an MB-preferential or KC subtype-preferential manner in the honeybee brain, suggesting that each KC subtype in the honeybee has distinct molecular and cellular characteristics (see [20] for a previous review). Sequencing of the whole honeybee genome has greatly advanced molecular biologic studies of the honeybee [21, 22]. A comprehensive search for genes preferentially expressed in honeybee OLs revealed the differential expression of one of the identified genes, termed *mKast* (*middle-type Kenyon cell-preferential arrestin-related protein*), in the MBs compared with other previously identified genes [23]. *mKast* is preferentially expressed not only in the OLs but also in a novel KC subtype, which we termed the ‘middle-type’ KCs (mKCs), in the MB calyces. This indicates that honeybee MBs actually comprise four KC subtypes: class-I lKCs, mKCs, and sKCs, and class-II KCs [23]. Careful discrimination of honeybee KC subtypes will require analysis of the function of each KC subtype by targeting genes expressed in an MB- and/or KC subtype-preferential manner in the honeybee brain.

In this review, we summarize recent progress in studies of brain region preferential gene expression patterns and neural activities in the honeybee brain, and discuss the possible roles of each KC subtype in the honeybee.

## Review

### Summary of genes expressed in a brain area-preferential manner in the honeybee brain

Based on the assumption that some brain regions are related to honeybee social behaviors and/or advanced brain functions, many groups have searched for genes that are expressed in a brain area-preferential manner in the honeybee brain (Table 1) (see [20] for a previous review).

**Table 1:** Summary of genes expressed in a brain area-preferential manner in the honeybee brain

Name	Function of the product	Worker brain area where preferentially expressed	Expression in queen and drone brains^a^	References
Calcium-signaling				
*IP*_*3*_ *R*	Inositol 1, 4, 5 (IP_3_)-trisphosphate receptor	lKC	W=Q=D	[24, 35, 36]
*CaMKII*	C^2+^/calmodulin-dependent protein kinase II	lKC	W=Q=D	[35, 36]
*PKC*	Protein kinase C	whole MB	N.A.	[35]
*IP*_*3*_ *P*	IP_3_ phosphatase	lKC	N.A.	[28]
*IP*_*3*_ *K*	IP_3_ kinase (Type A and B)	whole brain (/Type A, *96h > 48h > 0-1h*), and OL (/Type B, *0-1h > 48h = 96h*)^b^	N.A.	[47]
*Cac*	Calcium channel	MB *>* central brain	N.A.	[36]
*Ryr*	Ryanodine receptor	lKC	N.A.	[32, 36]
*Reticulocalbin*	Calcium-binding protein in the endoplasmic reticulum	lKC	N.A.	[32]
*PLCe*	Phospholipase C epsilon	whole MB	N.A.	[29]
Ecdysteroid/JH-signaling				
*Mblk-1*/*E93*	Ecdysone-regulated gene/ transcription factor	lKC	W=Q=D	[25]
*E74*	Ecdysone-regulated gene/ transcription factor	sKC	N.A.	[37]
*HR38*	Hormone receptor-like 38 (orphan receptor)	sKC, IIKC (*F > N*)	F>N=Q	[38]
*E75*	Ecdysone-regulated gene/ transcription factor	whole MB	N.A.	[30]
*BR-C*	Ecdysone-regulated gene/ transcription factor	lKC	N.A.	[30]
*USP*	Ultraspiracle (cofactor that binds EcR)	incKC (=lKC, *constitutive*) and icKC (=sKC, *1d > F*), and part of AL	N.A.	[38, 64]
*EcR*	Ecdysone receptor	sKC	N=F=Q	[39]
*JHDK*	JH diol kinase (enzyme that inactivates JH)	sKC and lKCs (but not mKC), IIKC^c^	N.A.	[33]
Other signaling				
*RJP-3*	Major royal jelly protein-3	‘A defined population of KCs’	N.A.	[40]
*PKA*	Catalytic subunit of cAMP-dependent protein kinase	lKC and sKC (entire inside of MB calyces)	N.A.	[36, 41]
*For* (*PKG*)	cGMP-dependent protein kinase	sKC and OL lamina (*F > N, preF > N*)	N.A.	[45]
*Mahya*	Secretory protein with a follistatin-like domain	‘small cell-body KCs’ and AL (*28d > 7d > NE*)	W=Q=D	[46]
*MESK2*	Protein implicated in Ras/MAPK-signaling	transverse zone in ventral OL	W=Q=D	[31]
*mKast*	mKC-preferential arrestin-related protein	mKC (but not lKC or sKC) and OL^d^	N.A.	[23]
*sgg*	Protein kinase, GSK 3-β	MB > central brain	N.A.	[36]
Neurotransmitters and their biosynthetic enzymes, receptors or transporters				
*Dop1*	Dopamine D1 receptor	whole brain (*NE > 15d*)^e^	N.A.	[43, 92]
*Tyr1*	Tyramine receptor	whole brain	N.A.	[93]
*EAAT* ^f^	Glutamate transporter	icKC (=sKC) and OL (*NE = 1h > 24h = F*)	N.A.	[94]
*Dop2*	Dopamine D2 receptor	‘small-cell bodied KC’ (=sKC; *constitutive*), ‘(large-cell bodied KC (=1KC;* F>NE=N*) ‘outer small-cell bodied KC	W=D [i.e., *older D > NE D*, (large-cell bodied KC)]	[42, 43]
*OA1*	Octopamine (OA) receptor	whole brain	N.A.	[95]
*Apisα2*	Nicotinic acetylcholine receptor α2-subunit	ocKC (=II KC), incKC (=lKC), a part of OL, AL and DL	N.A.	[49]
*Apisα7-1*	Nicotinic acetylcholine receptor α7−1-subunit	a part of ocKC (=II KC), incKC (=lKC), a part of OL, AL and DL	N.A.	[49]
*Apisα7-2* (GB17254)	Nicotinic acetylcholine receptor α7−2-subunit	ocKC (=IIKC), inner chiasma, a part of OL, AL and DL	N.A.	[49]
*5-HT*_*7*_	Serotonin (5-HT) receptor 7	whole brain	N.A.	[96]
*Trp*	Tachykinin-related peptide (neuromodulator)	sKC, lKC (but not mKC), IIKC, and some neurons in OL, AL and SOG	W=Q=D	[44]
*Gad*	Glutamic acid decarboxylase (GABA synthetic enzyme)	OL and AL (but not MB)	N.A.	[48]
*Dop3*	Dopamine D3 receptor	whole brain	N.A.	[97]
*GB12077*	Muscarinic acetylcholine receptor	MB *>* central brain	N.A.	[36]
Morphology of neurons				
*Futsch*	Microtubule-associated protein	OL monopolar cell	W=Q=D	[31]
*Tau*	Microtubule-associated protein (22C10 antigen)	OL monopolar cell	W=Q=D	[31]
*Syt14*	Synaptotagmin 14	lKC	N.A.	[29]
*Dlg5*	Disc large 5	lKC	N.A.	[29]
ncRNAs				
*Ks-1*	Function unknown	sKC, IIKC and some large somata neurons	W=Q=D (sKC, IIKC), D>W (between MB and OL)	[26]
*Nb-1*	Function unknown	subpopulation of octopamine-positive neurons (*N > F*)	N>F>Q	[27]
*mir-276*	miRNA	sKC, IIKC and OL	N=F=D>Q (sKC, IIKC),	[75]
			N=F=D=Q (OL)	

Note that, in most studies, *in situ* hybridization was used for gene expression analysis, while northern blotting [47], transcriptome analysis [36] and reverse transcription-polymerase chain reaction [43] were also used in some studies

Original Table from reference [20] was modified (a column for ‘Expression in queen and drone brains’ was newly added), updated (18 genes were newly added) and used

*Abbreviations*: *MB* mushroom body, *OL* optic lobe, *AL* antennal lobe, *DL* dorsal lobe, *lKC* class-I large-type KC, *mKC*, class-I middle-type KC, *sKC* class-I small-type KC, *II KC* class-II KC, *ocKC* outer compact KC, *incKC* inner non-compact KC, *icKC* inner compact KC. Terminologically, incKC = lKC, icKC = sKC, and ocKC = II KC, respectively

^a^Information for gene expression in queen and drones are shown, when they are available. W; worker, Q; queen, D; drone, NE D; newly emerged drone. N.A.; not analyzed. = means similar expression levels. < and > means higher expression in right than in left and *vise versa*, respectively

^b^Information for age/labor-dependent change in gene expression are shown in parenthesis in italic, when they are available. NE, newly emerged worker; N, nurse bee; F, forager; preF, precocious forager. 0-1h, 1d (24h), 48h, 96h, 7d, 15d and 28d indicate 0-1h-, 1d (24h)-, 48h-, 96h-, 7day, 15day-, and 28day-old worker, respectively. = means similar expression levels. < and > means higher expression in right than in left and *vise versa*, respectively

^c^More detailed information for restricted expression patterns are shown in parenthesis, when they are available

^d^Note that, in all cases except *mKast, Mblk-1*, *CaMKII*, *JHDK*, *Trp*, *Syt14* and *Dlg5*, mKCs were not discriminated from lKCs (incKCs) or sKCs (icKCs)

^e^Genes for some major neurotransmitter receptors are also listed in this Table as references, though they show rather uniform expression in the whole brain; i.e., *Dop1*, *Tyr1*, *OA1*, *Dop3* and *5-HT*_*7*_

^f^The terms ‘*Am*’ are omitted from gene names, which were used in the original papers, because only *Apis mellifera* genes are listed in this Table

To identify such genes, we used the differential display method [24–27], in combination with cDNA microarray [23, 28–31], proteomic analysis [32, 33] and matrix-assisted laser desorption/ionization-time of flight mass spectrometry [34]. These studies established that the two class-I KCs of the honeybee brain, the lKCs and sKCs, have distinct gene expression profiles.

To our knowledge, at least 19 genes are expressed in an MB-preferential manner in the honeybee brain: *inositol 1,4,5-trisphosphate receptor* (*IP*_*3*_*R*) [24, 35, 36], *Ca*^*2+*^*/calmodulin-dependent protein kinase II* (*CaMKII*), *protein kinase C* (*PKC*) [35, 36], *IP*_*3*_*phosphatase* (*IP*_*3*_*P*) [28], *ryanodine receptor* (*Ryr*) [32], *reticulocalbin* [32], *phospholipase C epsilon* (*PLCe*) [29], *mushroom body/large-type Kenyon cell-preferential protein-1* (*Mblk-1*)*/E93* [25], *E74* [37], *hormone receptor-like 38* (*HR38*) [38], *E75* [30], *Broad-complex* (*BR-C*) [30], *ecdysone receptor* (*EcR*) [39], *juvenile hormone diol kinase* (*JHDK*) [33], *royal jelly protein-3* (*RJP-3*) [40], *protein kinase A* (*PKA*) [36, 41], *dopamine receptor 2* (*Dop2*; in forager) [42, 43], *synaptotagmin 14* (*Syt14*) [29] and *disc large 5* (*Dlg5*) [29]. Of these, nine are expressed in an lKC-preferential manner (*IP*_*3*_*R* [24, 25, 28], *CaMKII* [35, 36], *IP*_*3*_*P* [28], *Ryr* [32], *reticulocalbin* [32], *Mblk-1/E93* [25], *BR-C* [30], *Syt14* [29] and *Dlg5* [29]), and four are expressed in an sKC-preferential manner (*E74* [37], *HR38* [38], *EcR* [39], and *Dop2* [46, 52; in newly emerged workers and nurse bees]), and five are preferentially expressed throughout the MB (*PKC* [35], *E75* [30], *PKA* [36, 41], *Dop2* [42, 43; in foragers], and *PLCe* [29]) (Table 1).

Of these five, *Dop2* is unique in that its KC-subtype preferential expression changes with the division of labor of workers: *Dop2* is preferentially expressed in the sKCs in newly emerged workers and nurse bees, while it is expressed in the whole MBs in foragers [42, 43]. *JHDK* [33] and *Tachykinin-related peptide* (*Trp*) [44] are both expressed preferentially in both the outer part of the lKCs (previously termed ‘L-1’ and ‘L-a’ lKCs, respectively [33, 44]) and the entire sKC, but not in the inner part of the lKCs (‘L-2’ and ‘L-b’ lKCs, respectively [33, 44]), suggesting that lKCs could be further classified into subpopulations based on their gene expression profiles. Later, the ‘inner part’ of the lKCs was determined to correspond to the newly identified mKCs [23].

Many genes are preferentially expressed not only in the MBs, but also in some other brain areas: i.e., *foraging* (*for*) [45], *Mahya* [46] and *mKast* [23] etc. Other genes are preferentially expressed only in other brain areas, including the OLs, but not in the MBs; i.e., *IP*_*3*_*kinase* (*IP*_*3*_*K*) *Type-B* [47], *misexpression suppressor of dominant-negative kinase suppressor of Ras 2* (*MESK2*) [31], *glutamate decarboxylase* (*Gad*) [48], *futsch*, and *tau* [31] (Table 1). Although no gene is reported to be preferentially expressed in class-II KCs, *Apisα7-2* [49] is expressed in class-II KCs in the MBs and in other brain regions.

### Identification of novel ‘middle-type’ KCs, which are characterized by preferential *mKast*-expression

Previous studies were based on the notion that honeybee MBs comprise only three types of KCs: class I-lKCs, sKCs and class II KCs. We recently identified a novel type of KC, however, that we termed mKCs, as described below [23].

**Fig. 2:**
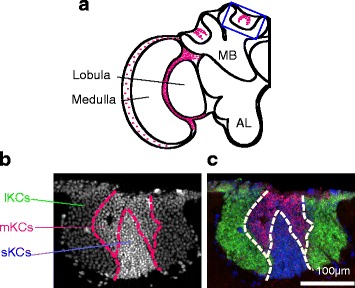
Identification of novel ‘middle-type’ KCs characterized by preferential *mKast-*expression. **a** Schematic drawing of the distribution of *mKast*-expressing neurons (magenta dots) in the left worker brain hemisphere. Note that the lamina of OL (the outermost layer) is absent in this illustration, which contains the entire MB structure, including the calyces and pedunculus. MB; mushroom body, AL; antennal lobe. The MB medial calyx boxed with blue line in panel (**a**) corresponds to both panels (**b**) and (**c**). **b** Nuclear staining with 4′,6-diamidino-2-phenylindole (DAPI) of class-I KCs localized inside of an MB calyx. Dashed lines indicate boundaries of the lKCs, mKCs and sKCs. **c** Double-fluorescence *in situ* hybridization for *CaMKII* (green), which is preferentially expressed in the lKCs, and *mKast* (magenta), which is preferentially expressed in the mKCs. Nuclei of the sKCs are counter-stained with DAPI (*blue*). Bar, 100 μm. For panels (**b**) and (**c**), an original figure from [23], which was published in *PLOS ONE*, an open access journal, was reused

Earlier studies indicated that honeybees gauge flight distance based on the optic flow they perceive during the foraging flight [50, 51]. As the honeybee OLs comprise distinct neuropil layers, the lamina, medulla and lobula, in which contrast, color and movement of the visual substance are processed, respectively (Fig. 2a) [52–55], we postulated that at least some sensory information obtained via optic flow is processed in the OLs, and thus applied a combination of differential display and cDNA microarray methods to search for genes that are highly expressed in honeybee OLs [23, 31]. Here we focus on one of the three identified genes, termed *mKast* (*middle-type Kenyon cell preferential-arrestin related protein*), which led to the discovery of the novel ‘middle-type’ Kenyon cells (Fig. 2) [23].

*mKast* (GB18367) encodes a predicted protein that has arrestin-like_N and arrestin-like_C domains, and low (~25%) sequence identity with mammalian arrestin domain-containing protein (ARRDC) 1–4 [23]. In addition to *mKast*, the honeybee genome contains three related genes encoding predicted proteins having 38%, 28% and 27% sequence identity with mKast, respectively. Interestingly, the genomes of some aculeate hymenopteran insects, such as the dwarf honeybee *Apis florea*, the bumblebee *Bombus terrestris*, the alfalfa leafcutter bee *Megachile rotundata*, and the parasitic jewel wasp *Nasonia vitripennis*, contain genes with higher sequence identity to *mKast* (97%, 85%, 82%, and 56%, respectively). Some other insect and invertebrate species contain genes that are less related to *mKast* (sequence identities <~30%), suggesting that *mKast* may be unique to aculeate hymenopteran insects [23].

**Fig. 3:**
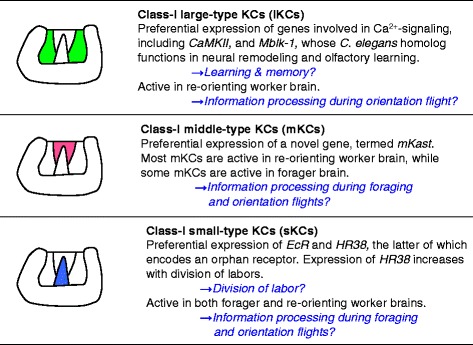
Summary of gene expression profiles and neural activities of honeybee class-I KC subtypes. Gene expression profile characteristic to the lKCs (*1* ^*st*^ line), mKCs (*2* ^*nd*^ line) and sKCs (*3* ^*rd*^ line) is described below the name of each KC subtype on each line. Assumed functions of each KC subtype are described after the arrows in each line. The left panels illustrate regions, in which the somata of each KC subtype are located, inside the MB medial calyx that is boxed with blue line in Fig. 2a. The lKCs (*1* ^*st*^ *line*), mKCs (*2* ^*nd*^ *line*) and sKCs (*3* ^*rd*^ *line*) are colored in *green*, *magenta* and *blue*, respectively. Original figure from [20] was modified (information regarding neural activity during orientation flight and a line for mKC are newly added) and used

**Fig. 4:**
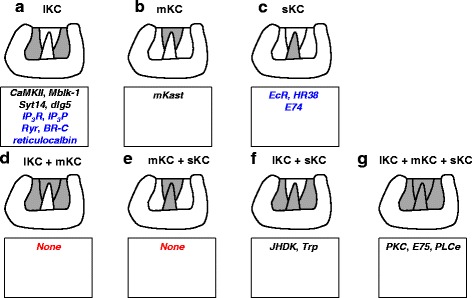
Possible expression patterns of genes in the honeybee MB calyx. (*Upper panels*) Possible combinations of class-I KCs expressing a certain gene in the honeybee MB calyx are illustrated in grey: expression in lKCs (**a**), mKCs (**b**), sKCs (**c**), lKCs + mKCs (**d**), mKCs + sKCs (**e**), lKCs + sKCs (**f**), and lKCs + mKCs + sKCs (**g**, the entire MB). (*Lower squares*) Genes that are expressed as illustrated by the upper panels are listed inside the squares below the panels. Genes whose expression pattern was confirmed by double *in situ* hybridization for *mKast* and the gene of interest (**a**, **b**, and **f**) or by single *in situ* hybridization (**g**) are written in *black*. Genes whose expression patterns were estimated from the previously reported results are written in *blue*. ‘None’ indicates that there are no corresponding genes whose expression was experimentally confirmed or can safely be estimated from the previous results

In the OLs, neurons with preferential expression of *mKast* are scattered in the lamina-medulla layer, whereas they are more widely distributed in the medulla-lobula layer (Fig. 2a). In contrast, the somata for neurons preferentially expressing mKast localize between the lKCs and sKCs in the MBs (Figs. 2 and 3). The MB area expressing *mKast* does not overlap with the MB areas expressing *CaMKII* or *Mblk-1*, which were originally considered to be preferentially expressed in the lKCs (Figs. 2b, 2c, 3 and 4) [25, 35, 36]. It is complementary with areas that express *Trp* or *JHDK*, which were originally reported to be preferentially expressed in the outer part of the lKCs and sKCs, but not in the inner part of the lKCs, which correspond to a novel KC subtype (Fig. 4) [33, 44]. We termed this novel KC subtype characterized by the preferential *mKast* expression mKCs, as the size of the mKC somata is intermediate between that of the lKCs (7–9 μm) and sKCs (5–7 μm), and the somata are localized between the lKCs and sKCs (Fig. 2b and c) [23].

### Functional categorization of genes preferentially expressed in honeybee MBs

As summarized in Table 1, various genes are expressed in an MB- and/or KC subtype-preferential manner in the honeybee brain. Interestingly, functional categorization of some of these genes implies that the lKCs, mKCs and sKCs have distinct functions in relation to honeybee behaviors and/or advanced brain functions. Here we provide examples of functional categorization of genes preferentially expressed in honeybee MBs, based on the current notion that honeybee MBs comprise four types of KCs: class-I lKCs, mKCs, sKCs and class-II KCs.

Five genes related to calcium signaling, which plays crucial roles in neurons involved in learning and memory [56–58], are preferentially expressed in the lKCs in the honeybee brain (Table 1, Figs. 3 and 4). Of these, *IP*_*3*_*R* [24, 35, 36] and *Ryr* [32] encode endoplasmic reticulum membrane Ca^2+^ channels, and *CaMKII* [35, 36] and *IP*_*3*_*P* [28] encode cytoplasmic enzymes related to calcium signaling. CaMKII senses and modulates synaptic activity responding to a high Ca^2+^ concentration [59], while IP_3_P dephosphorylates IP_3_, affecting IP_3_R opening and resultant Ca^2+^ concentration [60]. Reticulocalbin [32] is a calcium-binding protein localized in the endoplasmic reticulum. In addition, *PKC* [35] and *PLCe* [29] are preferentially expressed in the whole MBs. PKC is activated by diacylglycerol (DAG) [59]. Because IP_3_ and DAG are generated by phosphatidylinositol-phospholipid hydrolysis, which is catalyzed by PLC, both PKC and PLC are involved in calcium signaling. Learning and memory based on calcium signaling is conserved among animal species [56–58]. These findings suggest that synaptic plasticity based on calcium signaling is enhanced in the MBs, especially in the lKCs, in the honeybee brain (Fig. 3). There are, however, a few exceptions: *type-A IP*_*3*_*K*, which encodes an enzyme that terminates IP_3_ signaling [61], is expressed in the whole brain, whereas *type-B IP*_*3*_*K* is expressed in an OL-preferential manner (Table 1) [47]. Therefore, not all genes involved in calcium signaling are expressed in a MB-preferential or lKC-preferential manner.

In contrast, *EcR* is preferentially expressed in the sKCs in the honeybee brain (Table 1, Figs. 3 and 4) [39]. We previously showed that the gene for an orphan nuclear hormone receptor, *HR38*, is also preferentially expressed in the sKCs and at a high expression level in forager brains than in nurse bee brains [38]. In mosquito or *Drosophila*, HR38 responds to ecdysteroids other than ecdysone to induce a distinct set of target genes, unlike EcR [62, 63]. We previously proposed that the mode of ecdysteroid-signaling in the sKCs changes from EcR- to HR38-dependent, according to the division of labor of workers [38]. In contrast, *USP*, which encodes a co-factor of EcR and HR38, is expressed in the entire MBs in 1-day old workers, while its expression in the sKCs is selectively reduced in foragers (Table 1) [64]. Changes in the expression of *HR38* and *USP* in the sKCs may thus be related to the division of labor of workers and the interaction of HR38 with cofactors other than USP in forager sKCs.

Not all ecdysteroid-regulated genes, however, are expressed in an sKC-preferential manner in the honeybee brain. While *E74* is preferentially expressed in the sKCs (Table 1 and Fig. 4) [37], both *Mblk-1/E93* and *BR-C* are preferentially expressed in the lKCs [25, 30], and *E75* is preferentially expressed in the whole MBs in the honeybee brain [30]. In *Drosophila*, EcR is responsible for the activation of ecdysone-regulated genes, including *BR-C*, *E74*, *E75* and *Mblk-1/E93*, whereas Mblk-1/E93 is required for proper activation of *BR-C*, *E74*, and *E75*, which results in apoptosis of the larval salivary gland during metamorphosis [65]. Expression of some ecdysteroid-regulated genes that are preferentially expressed in the lKCs (*Mblk-1/E93* [25] and *BR-C* [30]) or whole MBs (*E75* [30]) may not be directly regulated by EcR or HR38, but rather by other transcription factors responding to ecdysteroid [64]. In nematode, MBR-1, an Mblk-1 homolog, functions in the pruning of excessive neurites during larval growth and is required for olfactory learning [66, 67]. Assuming that Mblk-1 functions similarly in the honeybee brain, these findings provide further support that lKCs are related to memory and learning in the honeybee brain (Fig. 3).

 We previously reported that genes for some enzymes involved in the latter stages of ecdysteroid synthesis [*Cytochrome P450* (*CYP*) *306A1*, *CYP302A1* and *CYP314A1*] are predominantly expressed in not only in the ovaries but also in the brain, suggesting that ecdysteroids are *de novo* synthesized in the brains of worker honeybees [68]. It is plausible that ecdysteroids synthesized in the worker brain regulate ecdysteroid-signaling via EcR or HR38 therein, like ‘neurosteroids’ in the vertebrates [69]. The role of ecdysteroids in the insect brain, in regulating memory [70], sleep [71] and the circadian clock [72], has recently attracted the interest of researchers [73]. In addition, olfactory aversive learning is modulated by ecdysteroid injection in the honeybee [74]. It is thus plausible ecdysteroids synthesized in the brain and ecdysteroid-signaling also function in other biologic phenomena in the honeybee.

### Expression of brain area-preferential genes in the queen and/or drone brains

How are these brain area-preferential genes expressed in the queen and/or drone brains? Of the 44 genes listed in Table 1, the expression of 14 (*IP*_*3*_*R* [35], *CaMKII* [35], *Mblk-1* [25], *HR38* [38], *EcR* [39], *Mahya* [46], *MESK2* [31], *Dop2* [42], *Trp* [44], *futsch* [31], *tau* [31], *Ks-1* [26], *Nb-1* [27], and *mir-276* [75]) has been studied in queen and/or drone brains. Of these 14 genes, the expression patterns of 11 (*IP*_*3*_*R* [35], *CaMKII* [35], *Mblk-1* [25], *EcR* [39], *Mahya* [46], *MESK2* [31], *Dop2* [42], *Trp* [44], *futsch* [31], *tau* [31] and *Ks-1* [26]) are similar in the MBs among worker, queen, and drone brains, suggesting that the major molecular characteristics of MB neurons are conserved in the honeybee brain irrespective of caste and sex. The expression profile of *Dop2* in the MBs changes similarly with age in both worker and drone brains [42]. The *HR38* expression is higher in the forager brain than in the nurse bee and queen brains [38], whereas *Nb-1* expression is high in the nurse bee brain, moderate in the forager brain, and low in the queen brain [27], suggesting their possible roles in modulating the division of labor and/or caste-dependent behaviors.

### Neural activities in forager and re-orienting worker brains and differentiation of KC subtypes in developing pupal brains

Some immediate early genes, such as *kakusei* homologs [76–78], *c-Jun* [79, 80], *Hr38* [81], and *Egr* [82, 83], have been used to map active brain regions in insects. Among them, some reports studied neural activities in the brains of worker honeybees that have experienced orientation or foraging flight. Neural activity was detected mainly in the whole MBs in the brains of re-orienting workers [76, 77, 82], whereas it was mainly detected in the center of the inside of the MB calyces in forager brains [76, 77, 83], indicating that distinct KC subtypes are mainly active in the brains of re-orienting workers and foragers. The mKCs, however, were not distinguished from the lKCs and sKCs in these studies. Our *in situ* hybridization experiments for *mKast*, an mKC marker, and *kakusei*, a neural activity marker, using serial sections of forager brains revealed some overlap among areas expressing *mKast* or *kakusei*; in addition to the entire sKCs, some mKCs were also active in the forager brains [23]. It is thus possible that, whereas all lKCs, mKCs, and sKCs play roles in information-processing during the orientation flight, sKCs and some mKCs may also play roles in information-processing during the foraging flight (Fig. 3).

How mKCs differentiate in the developing pupal brain during metamorphosis remains a question. Adult honeybee MBs develop from prepupal stage to pupal stage P9 (prepupal day to pupal nine days after puparium formation) during metamorphosis. Earlier studies indicated that both lKCs and sKCs are produced by a cluster of proliferating MB neuroblasts located at the inner core of the MB calyces during pupal brain development [84]. The lKCs, which are produced by MB neuroblasts at early pupal stages, are pushed out of the MB calyces and cease proliferating till the P3 stage, while the sKCs, which are subsequently produced at the middle pupal stages till the P6 stage, replace MB neuroblasts at the P5 to P7 stages. The expression of *mKast*, which characterizes mKCs, starts at the P7 stage in an area sandwiched between the lKCs and sKCs and its expression becomes more prominent at the P8 stage, suggesting that the mKCs begin to differentiate after the lKCs and sKCs stop proliferating [23]. These findings suggest that the mKC lineage branches from lKC and/or sKC lineage(s) by modifying its cellular characteristics.

The identification of the mKCs remains incomplete, however, because the morphology of this cell type has not yet been determined. Further characterization of the morphology of both the dendrites in the calyces and the axons in the peduncles and lobes of the mKCs by immunostaining with anti-mKast antibodies, Golgi-staining, or using genome-editing to insert *gfp* downstream of the *mKast* promoter would allow for investigation of the synaptic connections of these cells with specific classes of input/output neurons of the MBs and elucidation of their physiologic functions.

### How are mKCs discriminated from lKCs and sKCs?

Because mKCs have quite distinct molecular and cellular characteristics, it may be important to discriminate mKCs from lKCs and sKCs in future studies of honeybee KC subtypes. Currently, the only way to determine whether a gene of interest is expressed preferentially in mKCs is to compare gene expression patterns of *mKast* with the gene of interest using double-fluorescence *in situ* hybridization or single *in situ* hybridization in serial brain sections. Seven combinations of KC subtypes expressing the gene inside of the MB calyces (class-I KCs) can be imaged in the honeybee brain: preferential expression in 1) lKCs, 2) mKCs, 3) sKCs, 4) lKCs + mKCs, 5) mKCs + sKCs, 6) lKCs + sKCs, and 7) lKCs + mKCs +sKCs (the entire MBs) (Fig. 4).

As summarized in Fig. 4, the genes expressed in an MB and/or KC subtype-preferential manner in the honeybee brain can be categorized into the above seven expression patterns (Fig. 4a-g). There are 9, 1, 3, 0, 0, 2, and 3 genes that are safely categorized into each of the above seven categories based on their expression patterns reported previously (Fig. 4). It could be especially important to discriminate between patterns A and D as well as patterns C and E. Because the somata of the mKCs are localized between the lKCs and sKCs, if a gene of interest is expressed in both lKC and mKCs, the inner side of the expression area will appear to be thicker and extend more toward the inner core of the calyces than if the gene is expressed only in lKCs (Fig. 4a and d). If a gene of interest is expressed in both sKCs and mKCs, both the upper and lower sides of the expression area will appear expanded (Fig. 4c and e). *In situ* hybridization studies would then be recommended to confirm the assumption.

### Possible biological function of mKast and role of mKCs in the honeybee brain

Although mKast contains both arrestin-like_N and_C domains, it has no apparent sequence identity with honeybee arrestins. Instead, mKast belongs to a protein superfamily that comprises mammalian ARRDCs [23]. In mammals, arrestins comprise a protein family that regulates the signaling and trafficking of various G protein-coupled receptors [85, 86]. Recent studies revealed that mammalian arrestins and ARRDCs function in a hierarchical manner to traffic agonist-stimulated G protein-coupled receptors to sorting endosomes [87, 88]. For example, while β-arrestin2 (arrestin-3) functions as the primary adaptor that binds agonist-stimulated β_2_ adrenergic receptor (β_2_AR) to promote clathrin-dependent internalization, ARRDCs function as secondary adaptors that bind internalized β_2_AR complexes to traffic them to early endosomes [88]. Thus, mKast might also have a role in receptor regulation in neurons expressing *mKast*, including mKCs.

In contrast to the finding that both sKCs and lKCs are differentiated from the cluster of proliferating MB neuroblasts up to the P3 and P6 stages, respectively, *mKast*-expression begins at the P7 stage in the developing pupal brain [23]. It is thus plausible that mKCs differentiate from some lKC and/or sKC populations that have already ceased to proliferate. Considering that the preferential gene expression profile of mKCs is almost complementary to that of the lKCs and sKCs, and that preferential *mKast*-expression is unique to mKCs, it might be that the induction of *mKast* is somehow related to the establishment of the gene expression profile unique to mKCs.

**Fig. 5:**
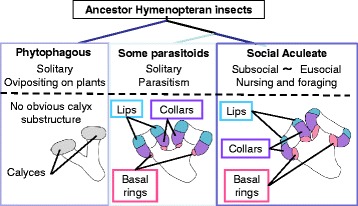
Illustration of the MB structure of some hymenopteran insects. (*Upper panel*) Schematic phylogenetic tree of the phytophagous bees, parasitoid wasps and social aculeate insects (honeybee) that derived from ancestor hymenopteran insect. (*Middle* and *lower panels*) Characteristics of the life history (*middle panels*) and schematic drawings of the MB structures of the phytophagous bees, parasitoid wasps and social aculeate insects (*lower panels*), respectively. Lips and collars of MB calyces in the parasitoid wasps and social aculeate insects are colored in blue and purple, and basal rings of MB calyces of a social aculeate insect (honeybee) are colored in pink, respectively. Original figures (photos) from [10] were modified and used with permission

Analysis of the molecular phylogenetic tree suggested that Aculeata Hymenoptera uniquely express *mKast* [23]. Thus, Aculeate Hymenoptera mKast homologs and mKCs have developed specific functions in the central nervous system during the evolution. Importantly, based on morphologic observations, it is reported that parasitoidism, and not sociality, is linked to the evolution of large and elaborate MBs in hymenopteran insect brains [10]. The authors proposed that the cognitive demands of host-finding behavior in parasitoids drove the acquisition of the evolutionarily novel MB architecture [10], as illustrated in Fig. 5. The discrimination of KC subtypes based on KC-subtype preferential gene expression in hymenopteran insects other than honeybees, however, has not yet been examined. Our findings suggest a possible relationship between the MB functions involving mKCs expressing *mKast* and foraging behaviors of the hymenopteran insect species. To test this hypothesis, it will be important to 1) perform functional analysis of *mKast* using reverse genetic methods, 2) analyze the projection patterns of mKCs in the honeybee brain, and 3) perform expression analysis of *mKast* homologs in the brains of various hymenopteran insects. The CRISPR/Cas9 method [89, 90] was recently demonstrated to be applicable to the honeybee [91], which will help to test the above hypothesis.

## Conclusions

Our findings demonstrate that honeybee MBs actually comprise four distinct KC subtypes: the previously known class-I lKCs and sKCs and class-II KCs; and novel class-I mKCs characterized by the preferential expression of *mKast*. The gene expression profile of mKCs is almost complementary to that of the lKCs and sKCs and the mKC lineage seems to branch from lKC and/or sKC lineages after the cessation of lKC and sKC proliferation during metamorphosis. Although mKCs exhibit quite unique molecular and cellular characteristics in comparison with lKCs and sKCs, the fact that the somata of the mKCs are localized between lKCs and sKCs, and that the size of the mKC somata is just intermediate of those of lKCs and sKCs makes it difficult to discriminate mKCs from lKCs and sKCs with precision in the honeybee brain based on their morphologies. Comparison of the expression areas of *mKast* with that of the gene of interest using *in situ* hybridization is currently the only method to precisely discriminate whether or not the gene of interest is expressed in the mKCs, but the unique localization of mKCs may help to solve this problem.

The biological function of mKast and the role of mKCs in regulating honeybee social behaviors, especially foraging behaviors, are intriguing subjects for future research. Given that mKast belongs to a protein superfamily that contains mammalian ARRDCs, it is plausible that mKast also functions to regulate receptor function in honeybee brain mKCs. In addition, considering that *mKast* is likely to be unique to the aculeate hymenopteran insects and that sKCs and some mKCs could be involved in information processing during the foraging flight, it will be intriguing to investigate the relationship between the molecular evolution of mKast/acquisition of mKCs and the functional and morphologic evolution of the MBs of hymenopteran insects.

## Abbreviations

5-HT_7_, 5-hydroxytryptamine (serotonin) receptor 7; AL, antennal lobe; ARRDC, arrestin domain-containing protein; BR-C, broad-complex; CaMKII, Ca^2+^/calmodulin-dependent protein kinase II; Cas, CRISPR associated protein; CRISPR, clustered regularly interspaced short palindromic repeat; CYP, cytochrome P450; DAG, diacylglycerol; Dlg5, discs large 5; Dop2, dopamine receptor 2; Dop3, dopamine receptor 3; EcR, ecdysone receptor; For, foraging; Gad, glutamate decarboxylase; GPCR, G protein-coupled receptor; HR38, hormone receptor-like 38; IP_3_, inositol 1, 4, 5-trisphosphate; IP_3_K, inositol 1, 4, 5-trisphosphate kinase; IP_3_P, inositol 1, 4, 5-trisphosphate phosphatase; IP_3_R, inositol 1, 4, 5-trisphosphate receptor; JHDK, juvenile hormone diol kinase; KC, Kenyon cell; lKC, large-type Kenyon cell; MB, mushroom body; Mblk-1, mushroom body/large-type Kenyon cell-preferential protein-1; MESK2, misexpression suppressor of dominant-negative kinase suppressor of Ras 2; mKast, middle-type Kenyon cell-preferential arrestin-related protein; mKC, middle-type Kenyon cell; OL, optic lobe; PKA, protein kinase A; PKC, protein kinase C; PLCe, phospholipase C epsilon; RJP-3, royal jelly protein-3; Ryr, ryanodine receptor; sKC, small-type Kenyon cell; Syt14, synaptotagmin 14; Trp, tachykinin-related protein; USP, Ultraspiracle
